# *Melastoma malabathricum* L. Suppresses Neutrophil Extracellular Trap Formation Induced by Synthetic Analog of Viral Double-Stranded RNA Associated with SARS-CoV-2 Infection

**DOI:** 10.3390/pathogens12020341

**Published:** 2023-02-17

**Authors:** Tse-Hung Huang, Pei-Wen Hsieh, Tsu-Jung Chen, Hui-Ju Tsai, Ju-Chien Cheng, Hsiang-Ruei Liao, Shun-Li Kuo, Ching-Ping Tseng

**Affiliations:** 1 Department of Traditional Chinese Medicine, Chang Gung Memorial Hospital, Keelung 204, Taiwan; 2 School of Traditional Chinese Medicine, Chang Gung University, Taoyuan 333, Taiwan; 3 Research Center for Chinese Herbal Medicine, Chang Gung University of Science and Technology, Taoyuan 333, Taiwan; 4 Graduate Institute of Health Industry Technology, Chang Gung University of Science and Technology, Taoyuan 333, Taiwan; 5 Graduate Institute of Natural Products, College of Medicine, Chang Gung University, Taoyuan 333, Taiwan; 6 Graduate Institute of Biomedical Sciences, College of Medicine, Chang Gung University, Taoyuan 333, Taiwan; 7 Department of General Surgery, Chang Gung Memorial Hospital, Chiayi 613, Taiwan; 8 Department of Medical Biotechnology and Laboratory Science, College of Medicine, Chang Gung University, Taoyuan 333, Taiwan; 9 Department of Medical Laboratory Science and Biotechnology, China Medical University, Taichung 404, Taiwan; 10 Department of Anesthesiology, Chang Gung Memorial Hospital, Taoyuan 333, Taiwan; 11 Division of Chinese Medicine Obstetrics and Gynecology, Department of Traditional Chinese Medicine, Chang Gung Memorial Hospital at Linkou, Taoyuan 333, Taiwan; 12 Graduate Institute of Clinical Medical Sciences, College of Medicine, Chang Gung University, Taoyuan 333, Taiwan; 13 Department of Laboratory Medicine, Chang Gung Memorial Hospital, Taoyuan 333, Taiwan

**Keywords:** double-stranded RNA, neutrophil extracellular trap, platelet aggregation, SARS-CoV-2 infection, traditional Chinese medicine

## Abstract

Platelet hyper-reactivity and neutrophil extracellular trap (NET) formation contribute to the development of thromboembolic diseases for patients infected with severe acute respiratory syndrome coronavirus 2 (SARS-CoV-2). This study investigated the pathophysiological effects of SARS-CoV-2 surface protein components and the viral double-stranded RNA (dsRNA) on platelet aggregation and NET formation. Traditional Chinese medicine (TCM) with anti-viral effects was also delineated. The treatment of human washed platelets with SARS-CoV-2 spike protein S1 or the ectodomain S1 + S2 regions neither caused platelet aggregation nor enhanced agonists-stimulated platelet aggregation. Moreover, NET formation can be induced by polyinosinic-polycytidylic acid (poly(I:C)), a synthetic analog of viral dsRNA, but not by the pseudovirus composed of SARS-CoV-2 spike, envelope, and membrane proteins. To search for TCM with anti-NET activity, the plant *Melastoma malabathricum* L. which has anticoagulant activity was partially purified by fractionation. One of the fractions inhibited poly(I:C)-induced NET formation in a dose-dependent manner. This study implicates that SARS-CoV-2 structural proteins alone are not sufficient to promote NET and platelet activation. Instead, dsRNA formed during viral replication stimulates NET formation. This study also sheds new insight into using the active components of *Melastoma malabathricum* L. with anti-NET activity in the battle of thromboembolic diseases associated with SARS-CoV-2 infection.

## 1. Introduction

Severe acute respiratory syndrome coronavirus 2 (SARS-CoV-2) is an enveloped positive single-stranded RNA virus and an emerging new coronavirus responsible for the transmission of the coronavirus disease 2019 (COVID-19) [1]. The RNA genome of SARS-CoV-2 is about 30,000 nucleotides in length [2]. Analysis of viral strains from infected patients indicate that SARS-CoV-2 was distinct from SARS-CoV-1, a deadly coronavirus that emerged in late 2002 and caused an outbreak of SARS [3], with about 79% identity in genome sequences [4]. The SARS-CoV-2 RNA genome is composed of 5′-terminal sequences, the central part sequences that are rich in open reading frame encoding sixteen non-structural proteins, and the 3′-terminal sequences encoding five structural proteins including spike (S), envelope (E), membrane (M), nucleocapsid (N), and hemagglutinin-esterase (HE) proteins [5]. The N protein holds the RNA genome, and the S, E, and M proteins together create the viral envelope. Among these structural proteins, spike is crucial for the infection of human cells by its capability to bind angiotensin-converting enzyme 2 (ACE2) at the surface of human target cells [6]. The ectodomain of the spike protein is composed of S1 and S2 subunits. The S1 subunit contains a receptor-binding domain which is important for ACE2 binding. The S2 subunit contains a fusion peptide and two heptad repeats that play a role in the formation of trimeric stalk and in the fusion of the viral and host membrane. The spike protein is thereby a potential therapeutic drug-target against SARS-CoV-2 infection [7].

In addition to the presentation of respiratory syndrome, patients with COVID-19 have an increased incidence of thromboembolic diseases such as pulmonary embolism, venous thromboembolism, and ischemic stroke [8,9]. Blood clots were found in almost every organ during autopsies on COVID-19 patients [10]. SARS-CoV-2 has been shown to potentiate agonists-induced platelet aggregation and increase platelet spreading on both fibrinogen and collagen [11], implying that platelet hyper-reactivity may contribute to COVID-19 pathophysiology. SARS-CoV-2 also induces neutrophil extracellular trap (NET) formation [12], which may cause tissue injury and induce inflammation and thrombosis when not properly regulated [13]. Understanding the underlying mechanisms of platelet hyper-reactivity and NET formation by SARS-CoV-2 facilitates the search for therapeutic regimens to reduce the incidence of thromboembolic diseases associated with SARS-CoV-2 infection.

Traditional Chinese Medicine (TCM) is a source for drug discovery against viral infection. Specific TCM formulas have been used or proposed for use in alleviating the respiratory syndrome associated with SARS-CoV-2 infection [14,15,16,17]. Not yet any TCM has been defined to reduce SARS-CoV-2-associated thromboembolic diseases. TCM with anti-platelet activity, anti-NET formation activity, or capability to improve blood circulation are potential candidates for developing therapeutic regimens to treat thromboembolic diseases. For example, baicalin inhibits the ACE2 pathway and platelet activation [18]. Polygonum multiflorum inhibits platelet aggregation without any effect on ACE2 [19]. Recently, casuarinin, a component of the plant *Melastoma malabathricum* L., has been identified as a neutrophil elastase inhibitor with a protective effect on chemotherapy-induced intestinal mucositis [20]. *Melastoma* spp. has multiple pharmacological effects with anticoagulopathy activity [21,22,23]. *Melastoma malabathricum* L. is therefore a good candidate to define whether it elicits anti-platelet or anti-NET formation activity.

In this study, the pathophysiological effects of SARS-CoV-2 surface protein components and the viral dsRNA on platelet aggregation and NET formation were investigated. Whether the plant components of *Melastoma malabathricum* L. elicited anti-platelet or anti-NET formation activity was also defined. The importance of this study for alleviating thromboembolic diseases associated with SARS-CoV-2 infection was discussed.

## 2. Materials and Methods

### 2.1. Materials

The SARS-CoV-2 (2019-nCoV) Spike S1-His recombinant protein containing SARS-CoV-2 spike protein S1 region (S1, Val16-Arg685), the SARS-CoV-2 (2019-nCoV) Spike S1 + S2 ECD-His recombinant protein containing the ectodomain of the S1 and S2 region (S1 + S2 ECD, Val16-Pro1213), and podoplanin (PDPN) were purchased from Sino Biological (Wayne, PA, USA). The high molecular weight polyinosinic-polycytidylic acid (poly(I:C)) was purchased from InvivoGen (San Diego, CA, USA). Collagen was purchased from Chrono-log (Havertown, PA, USA). Thrombin was purchased from Calbiochem (Darmstadt, Germany). U46619 was purchased from Cayman Chemical (Ann Arbor, MI, USA). Phorbol 12-myristate 13-acetate (PMA) was purchased from Sigma-Aldrich (St. Louis, MO, USA). Corning BioCoat 12 mm No. 1 German Glass Coverslips and the SYTOX Green nucleic acid stain reagent were purchased from ThermoFisher (Waltham, MA, USA).

### 2.2. Washed Platelet and Neutrophil Preparation

Washed platelets were prepared as described previously [24,25]. The peripheral blood (50 mL) from healthy volunteers was mixed with sodium citrate (3.15%). Platelet-rich-plasma (PRP) was collected by centrifugation at 740× *g* for 9 min. Platelets were then obtained by centrifugation of PRP at 980× *g* for 10 min in the presence of 0.5 μM prostaglandin I_2_. After washing twice with Tyrode’s buffer, the washed platelets were resuspended in Tyrode’s buffer containing Ca^2+^ and Mg^2+^ (1 mM MgCl_2_•6H_2_O and 2 mM CaCl_2_•2H_2_O).

On the other hand, neutrophils were prepared as described previously [26]. The peripheral blood was mixed with 3% dextran in the ratio of 1:1 and incubated at room temperature for 30 min. After the sedimentation of erythrocyte, the supernatant was collected for Ficoll gradient centrifugation at 4 °C and 400× *g* for 30 min. The cell pellets were then collected for the lysis of RBC in the RBC lysis solution (155 mM NH_4_Cl, 12 mM NaHCO_3_, and 0.1 mM EDTA) to remove erythrocyte contamination. After centrifugation at 4 °C and 200× *g* for 5 min, the neutrophils were resuspended in ice-cold HBSS/Ca^2+^ buffer before use.

### 2.3. NET Formation and Quantification

NET formation was performed as described previously with some modifications [26]. Briefly, freshly isolated neutrophils (10^5^/assay) were pre-incubated with either the test compound or solvent control for 5 min at 37 °C. The samples were placed on the poly-L-Lysine-coated coverslips and incubated at 37 °C for 3 h. After fixation with 2% paraformaldehyde (PFA), cells were stained with SYTOX Green nucleic acid stain reagent (2 μM), and NET formation was visualized with Zeiss Axiovert 200M immunofluorescence microscopy (Carl Zeiss, Jena, Germany). For the quantification of NET formation, the mounted coverslips were analyzed using ImageJ software.

### 2.4. Generation of Pseudovirus Expressing S, E, M Structural Proteins of SARS-CoV-2 (SEM Pseudovirus)

The SEM pseudovirus was provided by Dr. Chia-Yi Yu (National Institute of Infectious Diseases and Vaccinology, National Health Research Institutes, Miaoli, Taiwan). The construction and production of the pseudovirus have been described previously [27]. Briefly, the plasmids of helper pCMVΔR8.91, reporter pLKOAS3W-hyg^+^FLuc, and the cDNAs for S, E, and M structural proteins were co-transfected into 293T/17 cells by using the Lipofectamine 2000 Transfection Reagent (Invitrogen). At 24, 36, and 48 h after transfection, supernatants containing the pseudotyped lentiviruses were collected. Low-speed centrifugation was used to remove the cell debris present in the supernatants. The SEM pseudovirus was collected by filtration using a low protein-binding filter (0.45 μm) at 4 °C. The viral particle number was determined by real-time RT-PCR to quantify the RNA copies of the FLuc reporter gene.

### 2.5. Platelet Aggregation Assay

Platelet aggregation was performed as described previously [24,25]. Briefly, 495 μL washed platelet suspension (3 × 10^8^/mL) was added into a cuvette with continuous stirring at 37 °C for 1 min. The indicated agonists (5 μL) were added into the cuvette with continuous stirring at 37 °C for an additional 10 min. The aggregation status of platelets was monitored with a platelet aggregometer (Chrono-log) by measuring changes in light transmission.

### 2.6. Plant Material and Extraction of Melastoma malabathricum L.

Dried *Melastoma malabathricum* L. roots were purchased from Yuan-Feng (Zhongli, Taiwan). The species of the plant was confirmed by sequencing of the internal transcribed spacers of the genome which was performed by the Industrial Technology Research Institute (Hsinchu, Taiwan). The sequences were 100% similar (653 bp) to KY798016 in the NCBI GenBank database [20]. An extract from *Melastoma malabathricum* L. designated as MDN was obtained through extraction. Briefly, the materials (220 g) were refluxed twice with ddH_2_O (2200 mL) for 2 h. The resulting solution was filtered, and the filtrate was removed under a vacuum to harvest crude water extracts (MDN).

### 2.7. Statistical Analysis

Results and values were expressed as means ± S.E.M. of at least three independent experiments. Differences between control and treatment groups were evaluated by Student’s t-test or one-way ANOVA using the Prism statistical software version 4.0 (San Diego, CA, USA) when appropriate. Statistical significance was expressed as * *p* < 0.05 and ** *p* < 0.01.

## 3. Results

### 3.1. The Spike Protein of SARS-CoV-2 Does Not Trigger Platelet Aggregation

**Figure 1:**
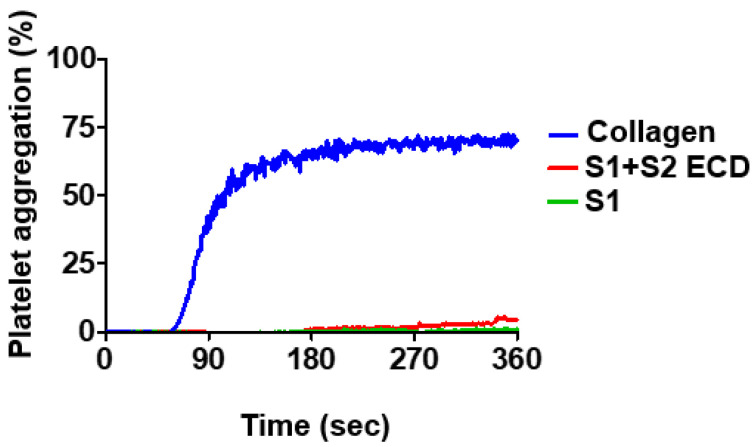
Effects of spike protein on platelet aggregation. Human platelets (3 × 10^8^/mL) were treated with collagen or the indicated recombinant spike protein (S1 + S2 ECD and S1) followed by analysis of platelet aggregation using the platelet aggregometer (Chrono-log). Representative traces of platelet aggregation are shown.

Patients with SARS-CoV-2 infection has been shown to present syndromes with hyperactive platelets [28]. The spike protein is responsible for virus entry to the host cells and contributes to SARS-CoV-2 infection by interaction with ACE-2 [29]. To investigate whether the spike protein causes platelet aggregation, human platelets were treated with the recombinant SARS-CoV-2 spike protein containing the S1 region (S1, Val16-Arg685) or the ectodomain of the S1 and S2 region (S1 + S2 ECD, Val16-Pro1213) followed by aggregation assay using a platelet aggregometer. In our analysis, neither S1 nor S1 + S2 ECD was able to stimulate platelet aggregation, while platelets responded normally to the stimulation by collagen (Figure 1). These data indicate that the spike protein alone is not sufficient to trigger platelet aggregation.

### 3.2. The Spike Protein of SARS-CoV-2 Does Not Enhance Agonist-Stimulated Platelet Aggregation

**Figure 2:**
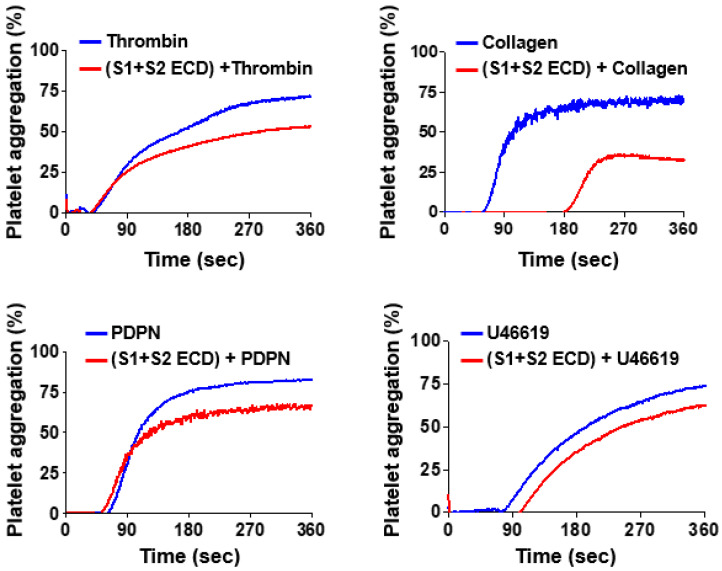
Effects of SARS-CoV-2 spike protein on agonists-stimulated platelet aggregation. Human platelets were pretreated with S1 + S2 ECD recombinant protein followed by treatment of platelet agonists thrombin, collagen, PDPN, and U46619, respectively. Platelet aggregation was analyzed by using the platelet aggregometer (Chrono-log). Representative traces of platelet aggregation curves are shown.

To investigate whether the spike protein of SARS-CoV-2 can enhance platelet aggregation stimulated by platelet agonists, human platelets were pretreated with the S1 + S2 ECD protein followed by treatment of the platelet agonists thrombin, collagen, PDPN, and U46619, respectively. A platelet aggregometer was used to monitor the status of platelet aggregation. Thrombin, collagen, PDPN and U46619 stimulated platelet aggregation as expected. However, the S1 + S2 ECD protein did not further enhance the degree of platelet aggregation. Instead, platelet aggregation was moderately inhibited by the S1 + S2 ECD protein (Figure 2). These data indicate that the spike protein does not further enhance agonist-stimulated platelet aggregation, and the protein alone may not be sufficient to induce thromboembolism in patients with SARS-CoV-2 infection.

### 3.3. Poly(I:C) but Not Pseudovirus with SEM Protein Induces NET Formation

**Figure 3:**
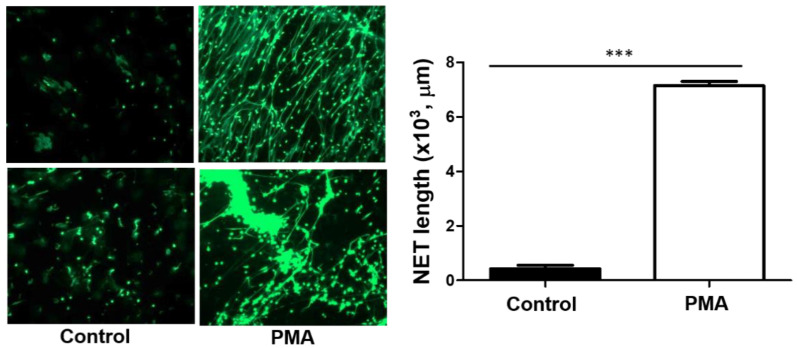
Establishment of method for NET formation analysis. Human neutrophils (10^5^/assay) isolated from healthy volunteers were placed on the poly-L-Lysine-coated coverslips for adhesion. After 30 min, neutrophils were incubated with PMA (100 nM) or its solvent control for 2 h at 37 °C. After fixation with 2% PFA, cells were stained with SYTOX Green (2 μM), and NET formation was visualized with Zeiss Axiovert 200 M immunofluorescence microscopy (Carl Zeiss, Germany). For quantification of NET formation, the mounted coverslips were analyzed using ImageJ software. Data represent the mean ± SEM of three independent experiments. ***, *p* < 0.001.

The aforementioned data did not support the direct effects of spike protein on platelet activation. NET formation has been shown to activate platelets and cause human washed platelets aggregation [13]. On the other hand, NETs contribute to immunothrombosis in COVID-19 patients with acute respiratory distress syndrome [12]. NETs can stimulate thrombosis in a platelet-dependent manner by the adhesion and activation of platelets and binding the cells to VWF and fibrinogen or by the direct activation of coagulation cascade [30]. Hence, NETs formation analysis was established in our laboratory in order to address the effects of SARS-CoV-2 protein and dsRNA components on NETs formation. After several testing, an optimal condition for NETs formation has been established. This is demonstrated by PMA-stimulated NET formation (Figure 3). A significant increase in NETs was observed after the treatment of human neutrophils with PMA.

**Figure 4:**
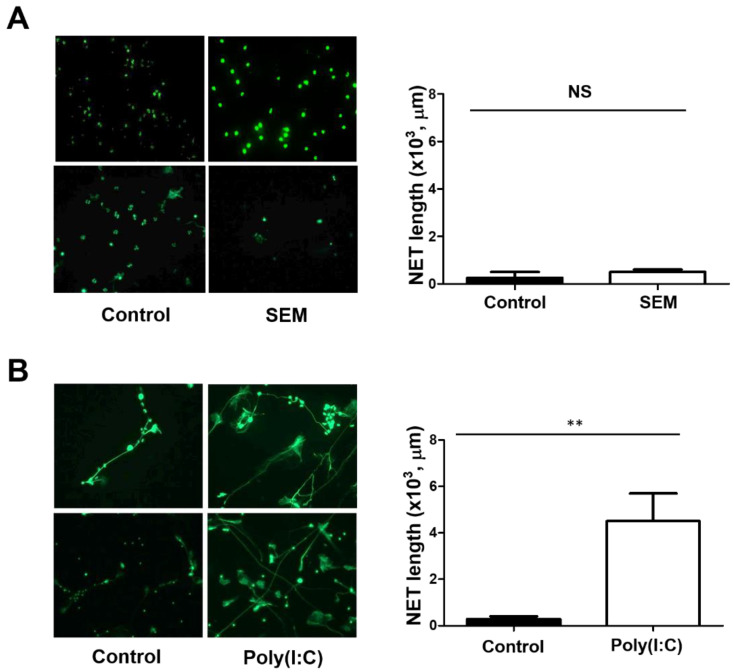
Poly(I:C) but not the SEM pseudovirus induces NET formation. Human neutrophils (10^5^/assay) isolated from healthy volunteers were placed on the poly-L-Lysine-coated coverslips for adhesion. After 30 min, neutrophils were incubated with the SEM pseudovirus (5 × 10^7^ copies) (**A**) or poly(I:C) (40 μg/mL) (**B**) for 2 h at 37 °C. After fixation with 2% PFA, cells were stained with SYTOX Green (2 μM), and NET formation was visualized by using Zeiss Axiovert 200M immunofluorescence microscopy (Carl Zeiss, Germany) (**A**,**B**). For the quantification of NET formation, the mounted coverslips were analyzed using ImageJ software. Data represent the mean ± SEM of three independent experiments. NS, not significant. **, *p* < 0.01.

We next investigated whether the pseudovirus containing the SARS-CoV-2 S, E, and M viral structural proteins without the RNA genetic component (SEM pseudovirus) can stimulate NET formation. Neutrophils were incubated with the SEM pseudovirus followed by NET formation assay. The SEM pseudovirus was not able to stimulate NET formation under our assay condition (Figure 4A). In contrast, poly(I:C), which was usually used to mimic dsRNA genetic components during RNA viral replication, was able to stimulate NET formation (Figure 4B). Together with a recent report showing that NETs promote inflammation and thrombosis [31], it is likely that an active virus with intact RNA genetic component is essential for NET formation by SARS-CoV-2.

### 3.4. The Extract of Melastoma malabathricum L. Inhibits Poly(I:C)-Induced NET Formation

According to the data presented in the previous section, the SARS-CoV-2 spike protein and other surface protein components are not sufficient to stimulate platelet aggregation and NET formation. The dsRNA formed during viral replication more likely plays a role in NET formation and platelet hyper-reactivity associated with SARS-CoV-2 infection. Hence, we searched for the compounds with suppressive activity on poly(I:C)-induced NET formation.

**Figure 5:**
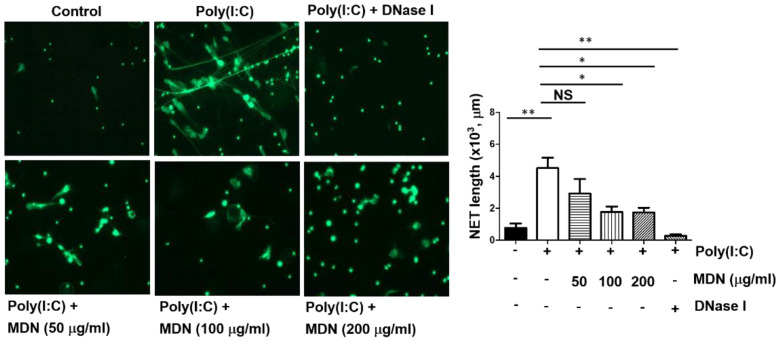
Extract of *Melastoma malabathricum* L. (MDN) suppresses poly(I:C)-induced NET formation. Human neutrophils (10^5^/assay) isolated from healthy volunteers were placed on poly-L-Lysine-coated coverslips for adhesion. After 30 min, neutrophils were pretreated with the indicated concentration of MDN or DNase I (1.25 U) for 5 min followed by incubation with poly(I:C) (40 μg/mL) for 2 h at 37 °C. After fixation with 2% PFA, cells were stained with SYTOX Green (2 μM), and NET formation was visualized by using Zeiss Axiovert 200 M immunofluorescence microscopy (Carl Zeiss, Germany). For quantification of NET formation, the mounted coverslips were analyzed using ImageJ software. Data represent the mean ± SEM of three independent experiments. NS, not significant. *, *p* < 0.05 and **, *p* < 0.01.

*Melastoma* spp. have multiple pharmacological effects with anti-inflammatory, hemostatic, anticoagulant, antioxidant, and hepatoprotective activities [21,22,23]. It has been used for the treatment of diarrhea, dysentery, leucorrhoea, ulcers and wounds [23]. An extract of *Melastoma malabathricum* L. (MDN) was obtained as a previous report, and it showed an inhibitory effect on human neutrophil elastase (HNE) with an IC_50_ value of 9.13 μg/mL [20]. Neutrophils were treated with MDN to investigate whether it can inhibit poly(I:C)-stimulated NET formation. We found that MDN inhibited poly(I:C)-stimulated NETs formation in a dose-dependent manner (Figure 5) and is a potential candidate against the development of thromboembolic diseases associated with SARS-CoV-2 infection.

## 4. Discussion

In the present study, we revealed that the spike protein and the SEM pseudovirus of SARS-CoV-2 are not able to stimulate platelet aggregation and NET formation. In contrast, viral mimetic poly(I:C) stimulates NET formation. MDN, an extract of *Melastoma malabathricum* L., elicits anti-viral activity by the suppression of poly(I:C)-stimulated NETs formation. This study provides new insight for the control of coagulopathy associated with SARS-CoV-2 infection.

Previous studies by Zhang et al. indicate that the spike protein of SARS-CoV-2 can stimulate platelet aggregation in the presence or absence of agonists [11]. Hemostasis and thrombosis are also highly active in patients with SARS-CoV-2 infection [32,33]. However, the findings of this study are not consistent with those previous reports. The discrepancies between these studies are not clear. It is likely that the spike protein of SARS-CoV-2 induces only moderate effects on platelet aggregation. The spike protein only enhances collagen-induced platelet aggregation by 10% [11]. Alternatively, active viral particles of SARS-CoV-2 are required for full activity in the stimulation of platelet aggregation and formation of thrombus. This may well explain why even the SEM pseudovirus of SARS-CoV-2 is not able to stimulate platelet aggregation in the ex vivo assays. Consistent with our observations, a recent study revealed that neither SARS-CoV-2 nor purified spike activates platelets [28]. Instead, the tissue factor released from infected cells is more likely to stimulate platelet activation in patients with SARS-CoV-2 infection. Rather than facilitating platelet aggregation, the spike protein was reported recently to reduce collagen-induced platelet aggregation [34], which is also observed in this study. The roles of the spike protein or other structural proteins on platelet hyper-reactivity therefore are still controversial. The underlying mechanisms for platelet activation in patients with COVID-19 remain to be elucidated.

dsRNA was formed during the replication of RNA virus. It is well known that the dsRNA is a molecular pattern associated with viral infection [35]. The synthetic analog poly(I:C) has been used to simulate the effects of viral infection, activate the transcription factors interferon regulatory factor 3 and NF-κB to initiate inflammatory response [36], and act as a pro-thrombotic and pro-coagulant molecule to disrupt the hemostasis balance on endothelial cells [31]. Recent studies revealed that NET formation was induced during RNA viral infection by interaction between pattern recognition receptors such as TLR3 and the viruses. SARS-CoV-2 has been shown to enhance NET formation, leading to the coagulopathy effects on patients with COVID-19 [37]. Although the spike protein and the SEM pseudovirus do not stimulate NET formation, the treatment of neutrophils with the viral mimetic poly(I:C) induces NETs formation. These findings implicate that the protein components of SARS-CoV-2 are not sufficient to activate neutrophils. Real viruses with replication capability to form dsRNA structures during the life cycle are required to cause NET formation.

Notably, we found that an extract of *Melastoma malabathricum* L. elicits anti-viral effects by the suppression of poly(I:C)-stimulated NETs formation. *Melastoma* spp. has been considered as a medicine to activate blood circulation and for the treatment of immune-related disorders [38]. Although the underlying mechanisms are not clear, the findings provide a venue to interfere with NET formation and likely reduce thromboembolic diseases associated with SARS-CoV-2 infection. MDN had been shown to suppress the activity of HNE, and HNE was able to trigger NET formation [26]. Whether MDN elicits inhibitory effects on poly(I:C)-induced NETs formation through inhibiting HNE and whether ACE2 expressing on the surface of leukocytes is involved in poly(I:C)-induced NETs formation are both worthy to investigate further.

Although we demonstrated that SARS-CoV-2 structural proteins are not sufficient to induce platelet activation, implying that a real virus or the RNA genetic components is crucial for platelet hyper-reactivity, limited access to real SARS-CoV-2 virus has restricted us from performing a more in-depth study. Moreover, NET is involved in inducing inflammation and thrombosis, and MDN from *Melastoma malabathricum* L. elicits inhibitory activity on poly(I:C)-stimulated NET formation. A study with real virus and/or the use of an animal model should be performed in future studies to gain comprehensive evidence before considering the use of MDN in the clinical setting in ameliorating thromboembolic complications associated with SARS-CoV-2 infection.

In conclusion, this study provides evidence that SARS-CoV-2 structural proteins alone are not sufficient to promote NET and platelet activation. Instead, dsRNA formed during viral replication stimulates NET formation. This study also shed new insight for using the extracts of *Melastoma malabathricum* L. with anti-NET activity in the battle of thromboembolic diseases associated with SARS-CoV-2 infection. A new strategy for alleviating COVID-19-associated abnormality in hemostasis and thrombosis is proposed that is worthy for further investigation.

## Data Availability

All relevant data are included in the paper.
